# A Combinatorial Reporter Set to Visualize the Membrane Contact Sites Between Endoplasmic Reticulum and Other Organelles in Plant Cell

**DOI:** 10.3389/fpls.2020.01280

**Published:** 2020-08-18

**Authors:** Tingting Li, Zhidan Xiao, Hongbo Li, Chuanliang Liu, Wenjin Shen, Caiji Gao

**Affiliations:** Guangdong Provincial Key Laboratory of Biotechnology for Plant Development, School of Life Sciences, South China Normal University, Guangzhou, China

**Keywords:** chloroplast, endoplasmic reticulum, membrane contact sites, mitochondria, plasma membrane, reporter

## Abstract

The membrane contact sites (MCSs) enable interorganelle communication by associating organelles at distances of tens of nanometers over extended membrane surfaces and serve to maintain cellular homeostasis through efficient exchange of metabolites, lipid, and calcium between organelles, organelle fission, and movement. Most MCSs and a growing number of tethering proteins especially those involved in mediating the junctions between endoplasmic reticulum (ER) and other organelles have been extensively characterized in mammal and yeast. However, the studies of plant MCSs are still at stages of infancy, at least one reason might be due to the lack of bona fide markers for visualizing these membrane junctions in plant cells. In this study, a series of genetically encoded reporters using split super-folder GFP protein were designed to detect the possible MCSs between ER and three other cellular compartments including chloroplast, mitochondria and plasma membrane (PM) in plant cell. By expressing these genetically encoded reporter in *Arabidopsis* protoplasts as well as *Nicotiana benthamiana* leaf, we could intuitively observe the punctate signal surrounding chloroplast upon expression of ER-chloroplast MCS reporter, punctate signal of ER-mitochondria MCS reporter and punctate signal close to the PM upon expression of ER-PM MCS reporter. We also showed that the ER-chloroplast MCSs were dynamic structures that undergo active remodeling with concomitant occurrence of chloroplast dysfunction inside plant cells. This study demonstrates that ER associates with various organelles in close proximity in plant cells and provides tools that might be applicable for visualizing MCSs in plants.

## Introduction

Eukaryotic cells are filled with various membranous organelles. The intracellular membrane confers a specific and relatively stable internal environment to each organelle and consequent compartmentation of the cytoplasm, while it also hinders the quick flow of proteins, lipid, ions, or other metabolites between different cellular compartments. Among these membranous organelles, the endoplasmic reticulum (ER) might be the largest one with a continuous network of tubules and cisternae that are widely extended in the cytoplasm (Hawes et al., 2015). ER plays essential cellular functions in protein and lipid biosynthesis, and in the regulation of calcium homeostasis. To meet the demand of exchange information and materials, ER needs to frequently communicate with almost all classes of membranous organelles inside the cell. Such kinds of communications are achieved through not only vesicular transport within endomembrane system or long-distance transported signaling molecules like calcium but also direct physical contacts termed membrane contact sites (MCSs) (Phillips and Voeltz, 2016). When observed under electron microscope, membranes of organelles at these MCSs are positioned in very close vicinity (usually 10–40 nm distance), but do not fuse (Phillips and Voeltz, 2016). A number of proteins called tethers localize to the contact sites and help to maintain the functional integrity of MCS, thereby bridging the ER and respective membranes of other organelles to modulate intracellular calcium dynamics and signaling, lipid exchange, and organelle position (Prinz, 2014; Eisenberg-Bord et al., 2016).

Growing research in animal and yeast have revealed that ER forms contact sites with numerous membranous organelles and the plasma membrane (PM) (Phillips and Voeltz, 2016). One of the best characterized MCS is mitochondria-ER contact sites (MERCs), which enables Ca^2+^ flux from the ER to mitochondria and the exchange of phospholipids between these two organelles (Yang et al., 2018). Three-dimensional (3D) structure of MERCs in the yeast established by electron microscopy (EM) and electron tomography indicate that the ER is wrapped around mitochondria (Friedman et al., 2011). A multiprotein complex called ER-mitochondria encounter structure (ERMES), which consists of four proteins Mdm10, Mdm34, Mdm12, and Mmm1, is well known to maintain the integrity of MERCs (Kornmann et al., 2009). Whereas in mammalian cells, MERCs are controlled by several different mechanisms like the homotypic fusion of mitochondrial fusion GTPase MFN2 or the interactions between mitochondrial porin voltage-dependent anion selective channel protein 1 (VDAC1) and the ER-localized Ca^2+^ channel IP3R (Szabadkai et al., 2006; Naon et al., 2016). Another kind of well-characterized ER-based membrane association site is the ER-PM contact sites (EPCSs) (Gallo et al., 2016). In mammal and yeast the EPCSs exist abundantly and function to modulate phospholipid metabolism and signaling between cortical ER and PM (Saheki and De Camilli, 2017). In yeast, lots of proteins like the integral cortical ER protein Ist2p, the ER-resident protein Scs2/22, the lipid binding protein Osh and three tricalbins Tcb1-3 have been identified to localize on the EPCSs and to regulate ER-PM association or lipid transfer between them (Stefan et al., 2011; Manford et al., 2012; Eisenberg-Bord et al., 2016). In mammal, EPCSs consists of three tricalbin homologs known as E-Syts for extended synaptotagmin, two Scs2/22 homologs, VAP-A and VAP-B, and the oxysterol-binding proteins ORPs (Giordano et al., 2013; Luo et al., 2019). Besides MERCs and EPCSs, the ER is also found to contact with several other organelles, such as Golgi apparatus, peroxisome, lipid droplet, endosomes in yeast and mammalian cells (Phillips and Voeltz, 2016).

In plants, one of the well-characterized MCSs is the ER-PM connection. Under the electron microscope, numerous ER-PM association sites that resembles EPCSs observed in yeast and mammalian cells can be seen all around the plant PM inner surface (Wang et al., 2017). The plant-specific NETWORKED actin-binding protein, NET3C, and phylogenetically conserved vesicle associated membrane protein-associated protein (VAP27) have been shown to interact with each other to form homo- or hetro-dimers to regulate the dynamics of EPCSs in plant (Wang et al., 2014; Wang et al., 2016). Actin filaments and microtubules seem to engage in the regulation of plant EPCSs, since NET3C and VAP27 associates with actin and tubulin respectively (Hawkins et al., 2014; Wang et al., 2014). Moreover, a phospholipid-binding protein Synaptotagmin1 (SYT1), the plant homolog of tricalbin/ESyts, has been shown to be a component of EPCS complex and define a different population of EPCSs, which are not overlap with those labeled by VAP27 or NET3C (Perez-Sancho et al., 2015; Wang et al., 2017). Another unique feature in plant is that one specific population of EPCSs overlaps with plasmodesmata (PD) as revealed by the PD associations with VAP27 and SYT1 (Levy et al., 2015; Wang et al., 2016). Besides EPCSs, another kind of MCS observed in plant is the ER-chloroplast contact sites also known as plastid-associated membranes, which are thought to be critical for lipid biosynthesis and homeostasis in plant cells (Liu and Li, 2019). Some lipid transporters like trigalactosyldiacylglycerol (TGD) family protein and lipid processing enzyme like the BnCLIP1 identified in *Brassica napus L* are found to potentially localize on the ER-chloroplast contact site (Tan et al., 2011). Until now, the molecular machinery or tethering proteins that regulate the contact between ER and chloroplast remain unknown. The ER are also found to associate with peroxisome and vacuole in plants, but whether these associations stand for the typical and stable contact site and how these associations are maintained remain elusive (Liu and Li, 2019).

Comparing with the extensive study of MCSs in mammal and yeast, the molecular basis of maintaining MCSs as well as the functions of these MCSs are still poorly understood in plants. One reason is that most tethering proteins mediating MCSs as identified in yeast and mammal do not have obvious homology sequences in plant. Another obstacle might be due to the lack of efficient tools or markers to quickly observe these MCSs in plant cells. In this study, we developed a series of reporters to visualize the potential MCSs between ER and three types of membranes including plasma membrane, mitochondria and chloroplast in plant cells.

## Materials and Methods

### Plant Materials

Seeds of *Arabidopsis thaliana* (Col-0) and tobacco (*Nicotiana benthamiana*) were surface sterilized and stratiﬁed at 4°C for 48 h in darkness before sowing on standard MS (Murashige and Skoog) plates supplemented with 1% sucrose and 0.8% agar at 22°C under long-day (LD) conditions (16-h light/8-h dark) for 5 days. The *Arabidopsis* plants were grown in a greenhouse at 22°C, while tobacco plants were cultivated at 28°C in greenhouse, both under LD conditions.

### Plasmid Construction

Each organelle targeting sequence used in this study was truncated form that only contains the sequence required for organelle targeting. UBC32 (At3g17000.1) contains the residues from 232-309 amino acid, OEP7 (At3g52420) contains the residues from 1–64 amino acid, OM64 (At5g09420) contains the residues from 1–60 amino acid, and LRR84A (At2g23300) contains the residues from 1-361 amino acid. All of these truncated fragments were PCR-amplified and separately inserted into protoplast transient expression vector pBI221-GFP to generate pBI221-GFP-UBC32, pBI221-OEP7-GFP, pBI221-OM64-GFP, and pBI221-LRR84A-GFP.

To construct pBI221-spGFP1-10-UBC32, full-length GFP sequence of pBI221-GFP-UBC32 was replaced by spGFP1-10 sequence. Similarly, the other half of the reporter was generated by the same way. To construct pBI221-OEP7-spGFP11, pBI221-OEP7-2XspGFP11, pBI221-OEP7-4XspGFP11, GFP sequence of pBI221-OEP7-GFP was respectively replaced by one, two, or four copies of spGFP11. As for pBI221-OEP7-GS-spGFP11, pBI221-OEP7-GS-2XspGFP11, pBI221-OEP7-GS-4XspGFP11, GFP sequence of pBI221-OEP7-GFP was replaced by GS-4XspGFP11, GS-spGFP11, GS-2XSPGFP11, or GS-4XSPGFP11. The GS linker sequence is “GSGSNGSSGGGSGGGSG”. Mitochondria related plasmids pBI221-OM64-spGFP11, pBI221-OM64-2xspGFP11, pBI221-OM64-4XspGFP11, pBI221-OM64-GS-spGFP11, pBI221-OM64-GS-2XspGFP11, pBI221-OM64-GS-4XspGFP11, and PM related plasmid pBI221-LRR84A-spGFP11, pBI221- LRR84A -2XspGFP11, pBI221- LRR84A -4XspGFP11, pBI221- LRR84A -GS-spGFP11, pBI221- LRR84A-GS-2XspGFP11, pBI221-LRR84A-GS-4XspGFP11, were constructed based on the same principle. To construct pBI221-VAP27-1-mCherry and pBI221-SYT1-mCherry, The PCR product was digested and ligated into the pBI221.

To generate plasmids used for transient transformation in tobacco leaf, the gene expression cassettes containing UBQ10 promoter, target gene and NOS terminator were PCR-amplified and introduced into binary vector pCAMBIA1300 by homologous recombination technology. All primers used in this study were provided in **Supplementary Table S1**.

### Transient Transformation

In *Arabidopsis* protoplast transient expression assay, the different plasmid combinations as indicated were transfected into mesophyll protoplasts derived from 3-week-old *Arabidopsis* plant. Then the transfected cells were incubated in the dark for 12 h before imaging. The leaf protoplast isolation and transient expression were performed as described previously (Li et al., 2019).

For tobacco leaves transient expression assay, the pCAMBIA1300 plasmids were transformed into *Agrobacterium tumefaciens* *GV3101* separately, and then co-infiltrated into the leaves of 5-week-old tobacco plants with MES buffer at OD_600_ of 0.5 (10 mM MgCl_2_, 10 mM MES pH 5.7, and 100 µM acetosyringone). The transfected plants were kept in the light for 2 days and the transfected epidermal cells were directly observed or exposed to different treatments. For methyl viologen (MV) treatment, the transfected tobacco leaves were further infiltrated with 100 μM MV for 5 h before imaging. For high light and dark treatment, the tobacco leaves with *Agrobacterium* filtration for 2 days were subjected to either high light exposure (1200 μmol m^-2^ s^-1^ detected on the leaf surface) for 4 h or darkness for 24 h, followed by confocal observation.

### Fluorescence Microscopic Observation

Imaging was captured with Zeiss LSM 800 confocal laser scanning microscope and Leica Upright Fluorescence Microscope DM6. The GFP signal was visualized with excitation at 488 nm and emission at 500 to 550 nm and mRFP1/mCherry signals were visualized in another detection channel with excitation at 559 nm and emission at 570 to 630 nm. Line-sequential scanning mode was always selected in dual-channel observation to avoid the possible cross talk between two ﬂuorophores. Confocal microscopy images were processed using the Zeiss ZEN, and all images were prepared using Adobe Photoshop.

## Results

### Designing a Series of Reporters for Detecting MCSs of ER-Chloroplast, ER-Mitochondria, and ER-PM

**Figure 1:**
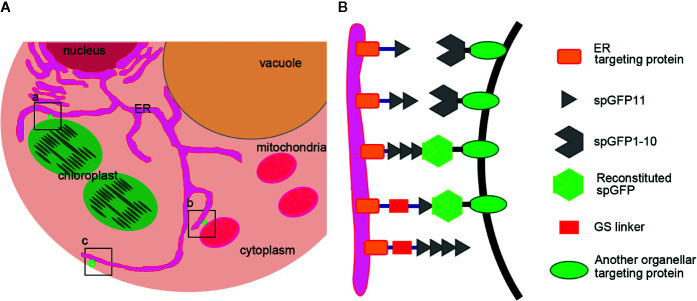
Design a series of reporters for MCSs of ER-Chloroplast, ER-Mitochondria and ER-PM. **(A)** A diagram shows where the reporters work. a) ER-Chloroplast membrane contact site, b) ER-Mitochondria membrane contact site, c) ER-PM membrane contact sites. **(B)** Schematic representation of the MCSs reporters. spGFP1-10 was anchored at the ER surface by fusing spGFP1-10 fragment with an ER membrane localization sequence. The spGFP11 was fused with another organelle targeting protein. Linkers (~3 nm) were inserted between spGFP11 and organelle targeting sequence to ensure that spGFP11 and spGFP1-10 could reach each other. When two organelles are close enough, spGFP1-10 and spGFP11 will refold and emit green fluorescence.

**Figure 2:**
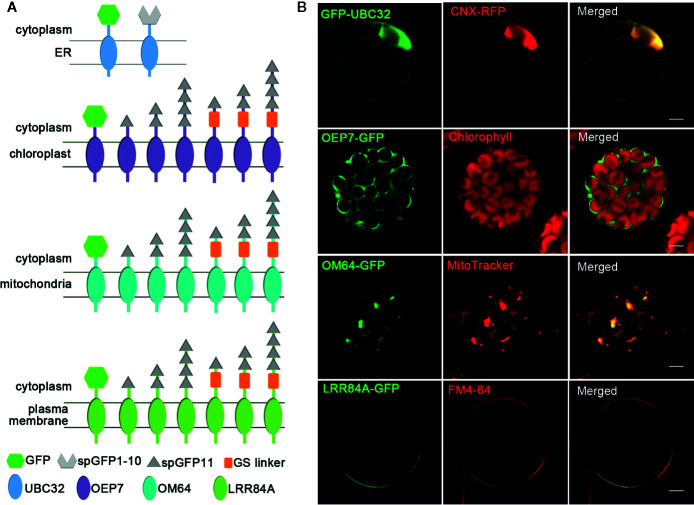
Schematic of MCSs reporter elements and the localization of four organelles membrane targeting fragments. **(A)** Schematic of fusion proteins used for detecting MCSs. The different fragments are shown in different shapes. **(B)** The localizations of four organellar membrane targeting fragments. The ER was marked by CNX-RFP; the chloroplast was detected by the autofluorescence of chlorophyll; mitochondria was stained by fluorescent dye MitoTracker Red CMXRos; PM was stained by FM4-64. Scale bar: 5 μm.

To create reporters that facilitate microscopic observation of MCSs, we used super-folder GFP as the key element to build fluorescent reporters of membrane junctions. We labeled the ER membrane and another organellar membrane with two parts of the split super-folder (sp) GFP protein, spGFP1-10 and spGFP11, respectively (Pédelacq et al., 2006; Pedelacq and Cabantous, 2019). If the two organelles are close enough, spGFP1-10 and spGFP11 will form functional spGFP and display fluorescent signal (**Figure 1**). This spGFP-based reporter system has been successfully used to visualize MCSs in yeast and mammalian cells (Eisenberg-Bord et al., 2016; Cieri et al., 2018; Yang et al., 2018). In *Arabidopsis*, the ubiquitin conjugase UBC32 is distributed on the ER membrane (Cui et al., 2012). We fused spGFP1-10 to a truncated form of UBC32 (residues 232-309) with deletion of the large N-terminal cytosolic part, whose N-terminus faces the cytosol and C-terminus harbors an ER anchoring transmembrane domain. Following the same principle, the spGFP11 was fused with fragments from OEP7 (residues 1–64) that is an outer envelope membrane protein located in chloroplast (Bae et al., 2008), a fragment of OM64 (residues 1–60) that is a mitochondria-bound outer membrane protein (Chew et al., 2004), and a fragment of PM localized membrane protein LRR84A (residues 1–361) (Cai et al., 2012), thereby anchoring spGFP11 fragment to the surfaces of chloroplast, mitochondria, and intracellular side of PM, respectively (**Figure 2A**). Full length GFP was also fused to these protein fragments to test whether they still display their correct subcellular localizations to respective cellular compartments. As expected, the resulting GFP-UBC32 fusion protein showed ubiquitous ER distribution and co-localized with the ER marker calnexin (CNX)-mRFP1 (Gao et al., 2012). Likewise, OEP7-GFP evenly distributed on the surface of chloroplast. OM64-GFP showed localization to the mitochondria as labeled by MitoTracker Red CMXRos, and LRR84A-GFP localized to the PM revealed by staining with FM4-64 (**Figure 2B**).

Since MCSs are spaced at 10 to 30 nm as demonstrated previously (Csordás et al., 2006), GS linker (~3 nm) was inserted between spGFP11 and other three cellular localization proteins including OEP7, OM64, and LRR84A to ensure that spGFP11 and spGFP1-10 could contact each other at the MCSs. Furthermore, in order to find the most appropriate MCSs reporter with bright enough signal intensity, we fused these chloroplast, mitochondrial and PM targeting sequences with one copy, two copies, or four copies of spGFP11, and designed a series of fusion protein with different numbers of spGFP11 copies and GS linker. The resulting constructs are summarized in schematic diagram (**Figure 2**).

### Characterization of the Reporter of ER-Chloroplast MCS

**Figure 3:**
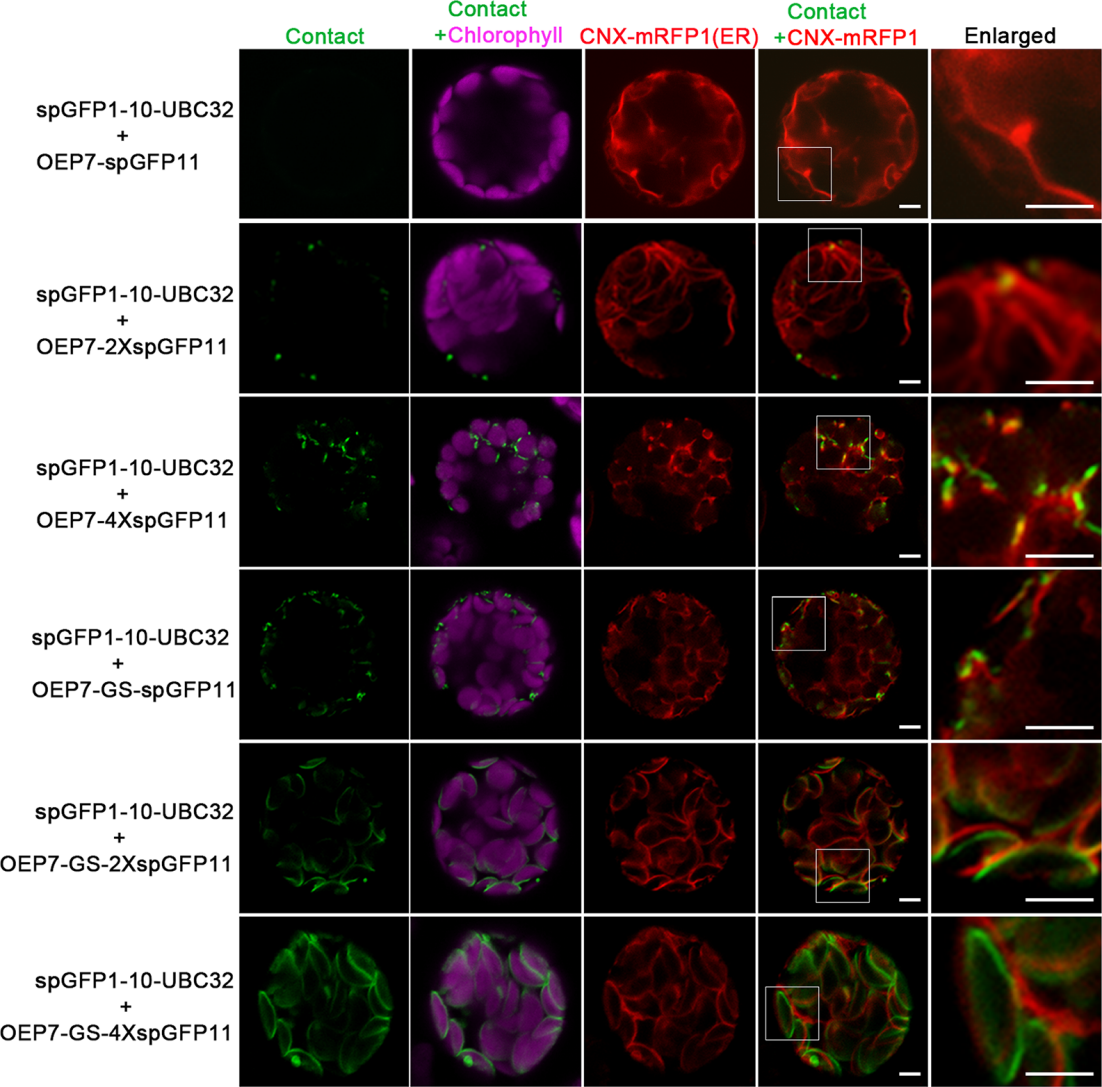
Characterization of the reporter of ER-chloroplast MCS in *Arabidopsis* protoplasts. *Arabidopsis* protoplasts were transiently transformed with different combinations of ER-chloroplast MCSs reporters followed by confocal observation. The contact sites were indicated by the green signals, the chloroplast was detected by chlorophyll autofluorescence, and ER was marked by ER marker CNX-RFP. Scale bar: 5 μm.

To test whether the reporter can detect the MCSs between ER and chloroplast, the spGFP1-10-UBC32 was co-expressed with various OEP7-spGFP11-fragment fusions in the *Arabidopsis* protoplasts and tobacco leaf cells. When spGFP1-10-UBC32 was co-expressed with OEP7-spGFP11, this combination did not yield any fluorescent signal (**Figure 3**). That is probably because the distance across the ER-chloroplast interface is more than the length of this reporter. As a consequence, the spGFP1-10 and spGFP11 cannot contact each other. With 2XspGFP11, a few puncta close to the chloroplast were observed, these puncta were also close to the ER marker CNX-mRFP1 (**Figure 3**). As the length of protein increased to 4XspGFP11, numerous brighter green signals with larger size around the chloroplast were observed in cells. Similar result was obtained in the combination of spGFP1-10-UBC32 and OEP7-GS-spGFP11 containing one GS linker. These punctate localization pattern resembles the pattern of MCSs observed in yeast, mammalian and plant cells (Tan et al., 2011; Wang et al., 2014; Yang et al., 2018), suggesting that they might reflect the MCSs of ER-chloroplast. As expected, the combination of spGFP1-10-UBC32 with OEP7-GS-2XspGFP11 with a longer extended length of spGFP11 showed much more stretched and much brighter signals as comparing with those fusions only containing repeated spGFP11 or one GS linker fused to one spGFP11 (**Figure 3**). These stretched and crescent signals surrounded the chloroplast with bright fluorescence. When OEP7-GS-4XspGFP11 was came into use, the widespread green signal was observed to fully wrap around chloroplast. These kinds of stretched signals with crescent or circular shape surrounding chloroplast obtained by the last two reporters suggest that these two reporters might possess too long extended spacer to reflect the true ER-chloroplast MCSs, because in these two cases the spGFP11 and spGFP1-10 can reach each other not merely at the narrow contact sites (**Figure 3**). To get an overall view of signal distributions of the above reporters inside protoplasts, we also observed the fluorescent signals under a wide-field fluorescent microscope and found a similar result as that shown in the confocal observation, which demonstrated that the reconstituted spGFP signals from the reporter with longer spacer length (e.g. OEP7-GS-4XspGFP11) became stretched to surround the whole chloroplasts (**Figure S1**). These results suggest that the ER-chloroplast MCS can be presumably detected by our reporter, and proper size of the reporter is important to obtain the correct observation of MCSs.

**Figure 4:**
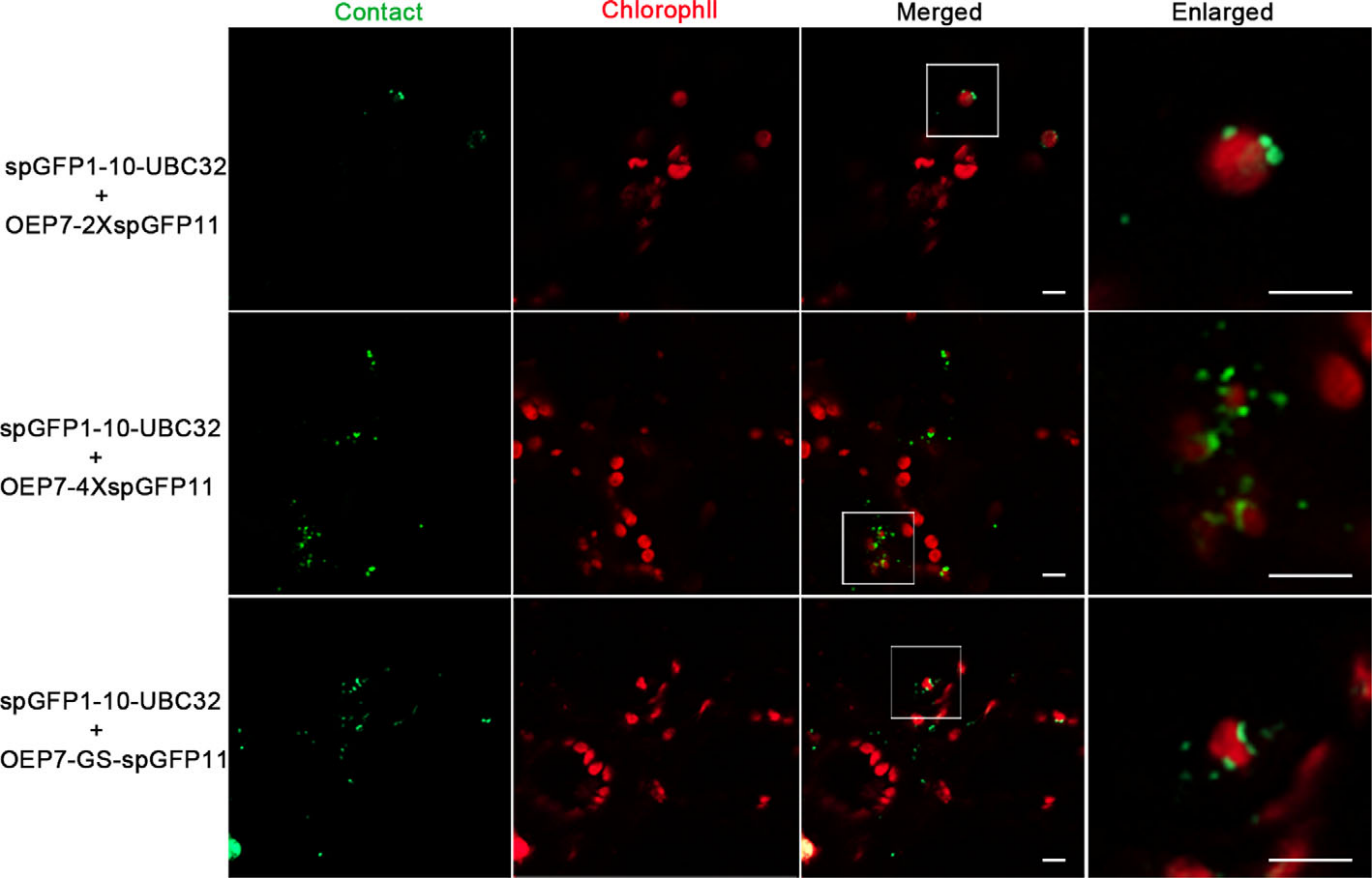
Characterization of the reporter of ER-chloroplast MCS in tobacco leaves. Different combinations of ER-chloroplast MCSs reporters were transiently expressed in tobacco (*N. benthamiana*) leaves followed by confocal observation. The MCSs were marked by the refold GFP, the chloroplast was detected by chlorophyll autofluorescence. Scale bar: 5 μm.

In order to further validate this system, the same experiments were performed in tobacco leaf. Similarly, the punctate GFP signals were found to localize around the chloroplast and the number of punctate signals increased as the adding of spGFP11 copies and GS linker (**Figure 4**). The combination of OEP7-2XspGFP11 and spGFP1-10-UBC32 generated bright but sparse signal around the chloroplast surface. When OEP7-4XspGFP11 was used, more fluorescent signals surround the chloroplast were observed (**Figure 4**). Similar results were obtained when OEP7-GS-spGFP11 was co-expressed with spGFP1-10-UBC32. The results obtained in tobacco leaf were basically consistent with those in *Arabidopsis* protoplasts. Considering the punctate signal distribution is more uniform in the combinations of either OEP7-GS-spGFP11 or OEP7-4XspGFP11 with spGFP1-10-UBC32, these two reporters might be more suitable for investigating ER-chloroplast MCSs in future work.

### Characterization of the Reporter of ER-Mitochondria MCS

**Figure 5:**
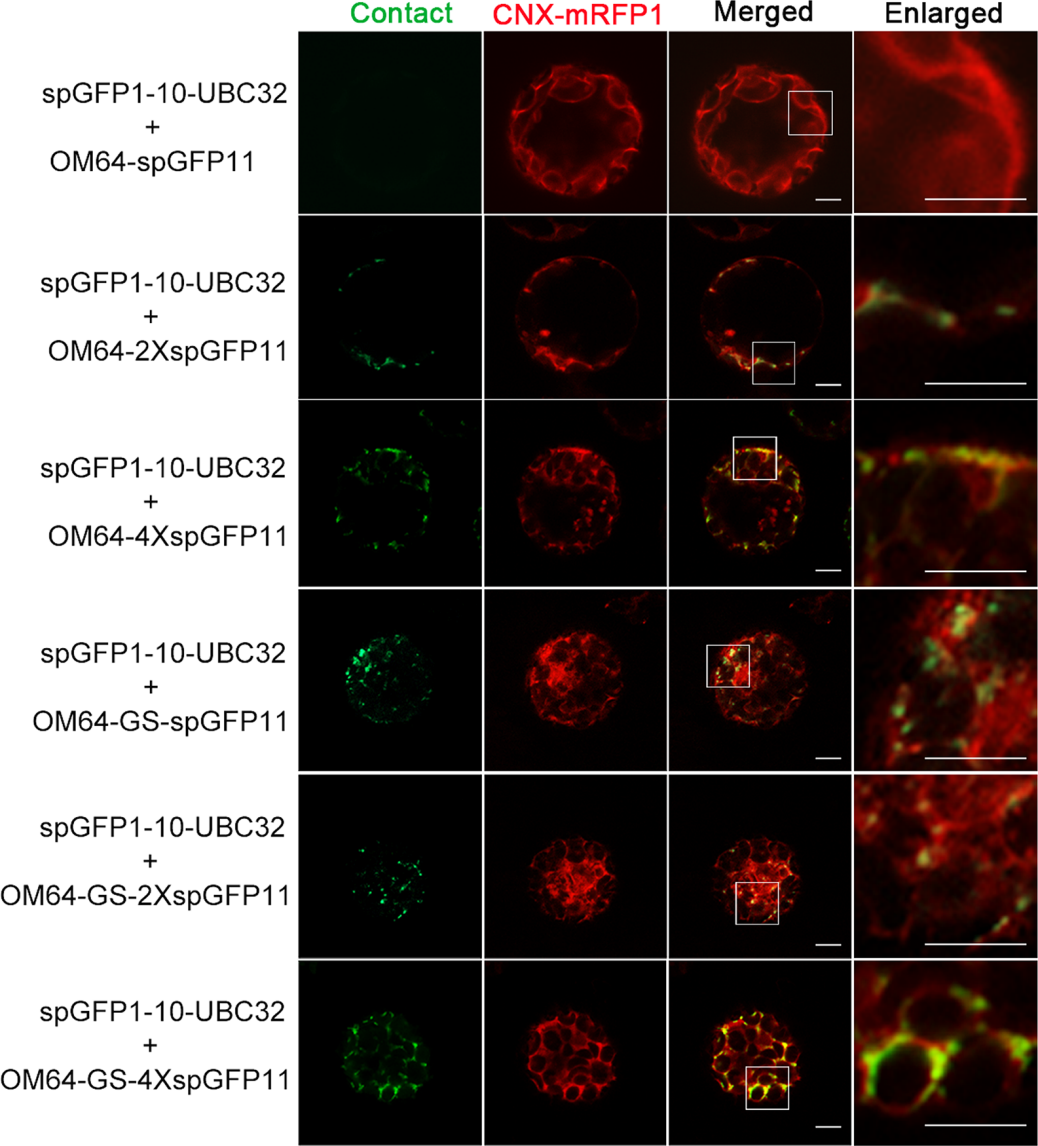
The reporter of ER-mitochondria MCS in *Arabidopsis* protoplasts. *Arabidopsis* protoplasts were transiently transformed with a series ER-mitochondria MCSs reporter and CNX-RFP followed by confocal observation. The MCSs were indicated by the green signals and ER was marked by ER marker CNX-RFP. Scale bar: 5 μm.

**Figure 6:**
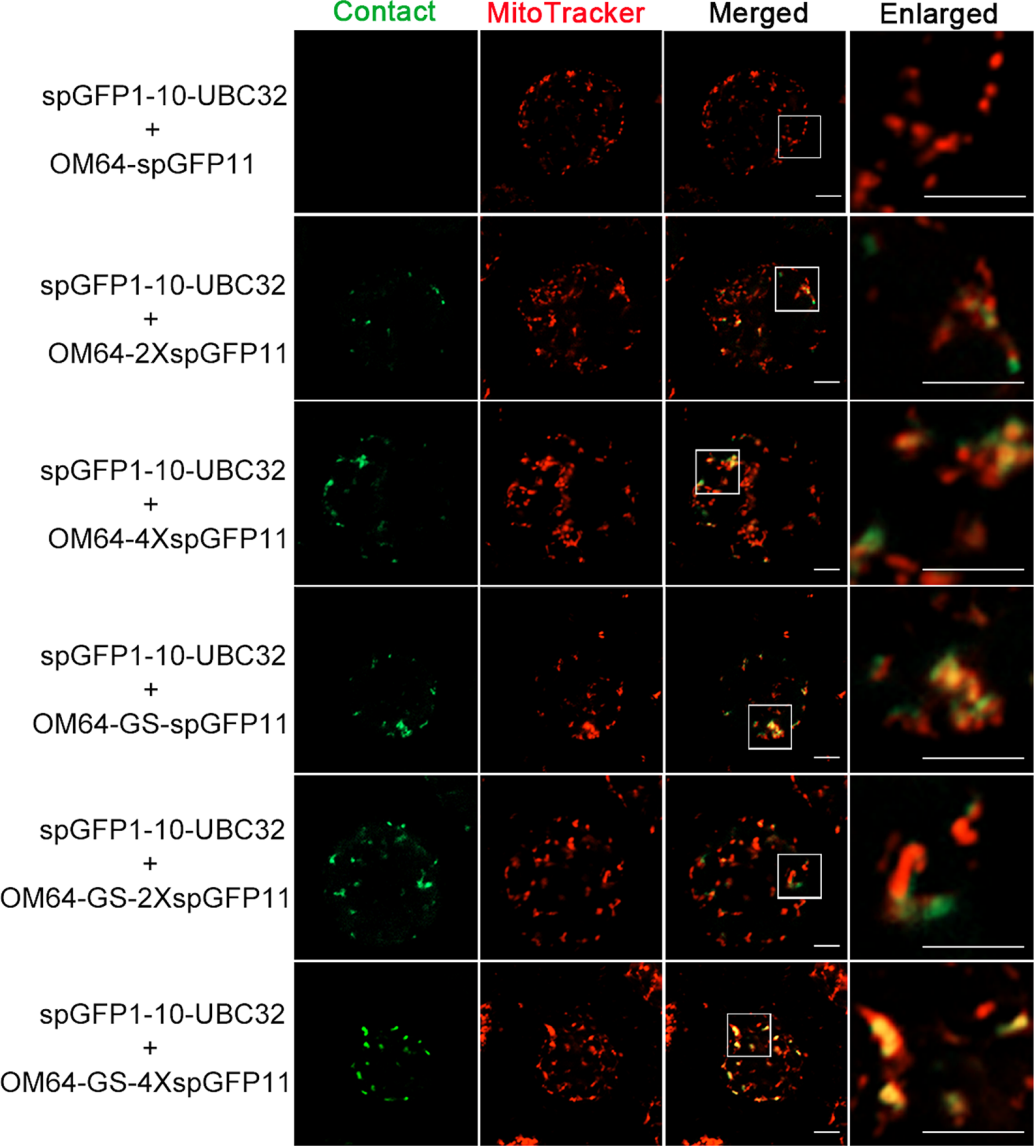
Colocalization of ER-mitochondria MCS reporter with mitochondria in *Arabidopsis* protoplasts. *Arabidopsis* protoplasts were transiently transformed with a series ER-Mitochondria MCSs reporters followed by confocal observation. The contact sites were indicated by the green signals; the Mitochondria were stained by MitoTracker Red CMXRos. Scale bar: 5 μm.

**Figure 7:**
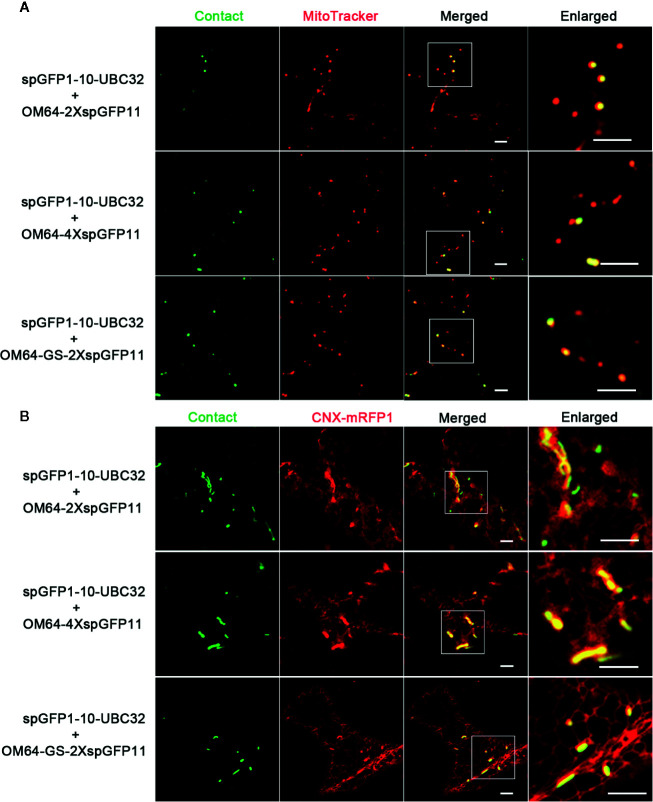
Characterization of the reporter of ER-mitochondria MCS in tobacco (*N. benthamiana*) leaves. **(A)** Tobacco (*N. benthamiana*) leaves were transiently transformed with a series ER-mitochondria MCSs reporter followed by confocal observation. The contact sites were indicated by the green signals; the mitochondria were stained by MitoTracker Red CMXRos. Scale bar: 5 μm. **(B)** Tobacco (*N. benthamiana*) leaves were transiently transformed with a series ER-mitochondria MCSs reporter followed by confocal observation. The contact sites were indicated by the green signals. The ER was marked by ER marker CNX-RFP. Scale bar: 5 μm.

To test if mitochondria can also form contact sites with ER in plant cells, we performed the same experiment to test the ER-mitochondria MCSs reporter in *Arabidopsis* leaf protoplasts. The obtained confocal data showed that the combination of spGFP1-10-UBC32 and OM64-spGFP11 did not yield any fluorescent signal (**Figures 5** and **6**). Some faint GFP signal emerged when spGFP1-10-UBC32 and OM64-2XspGFP11 was co-expressed. OM64-4XspGFP11 displayed more and brighter punctate signal. These punctate GFP signals partially overlapped with both ER and mitochondrial markers (**Figures 5** and **6**). Similar results were found when OM64-GS-spGFP11 or OM64-GS-2XspGFP11 was co-expressed with spGFP1-10-UBC32. Similar to the case in reporters of ER-chloroplast MCSs, the combination of spGFP1-10-UBC32 and OM64-GS-4XspGFP11 generated stronger signals with an enlarged area containing both ER and mitochondrial markers, indicating that OM64-GS-4XspGFP11 might have too long of an extended size for the study of ER-mitochondria MCS. When observed under wide-field fluorescent microscope, the reporter of OM64-GS-4XspGFP11 basically produced stronger signals comparing with those obtained from the shorter ones including OM64-GS-spGFP11 and OM64-GS-2XspGFP11 (**Figure S2**). These results are in agreement with the previous study, in which multiple copies of spGFP11 also displayed much brighter signals in their reporter used for detecting MERCs in mammalian cells (Yang et al., 2018). When expressed in tobacco leaf, the reconstituted GFP signals partially overlapping with mitotracker and ER marker could be found in the combination of OM64-2XspGFP11 and spGFP1-10-UBC32 (**Figures 7A, B**). With the increase of reporter size, the number of GFP puncta increased accordingly as revealed by more green signals overlapping with the mitotracker as shown in **Figure 7A**. The irregular or elongated GFP signals as observed in the top layer of the leaf epidermal cells might be due to the dynamically mitochondrial fission and fusion at the time point of taking images (**Figure 7B**). Collectively, these results suggest that the above obtained reporters can be presumably used to detect MERCs in the plant cell, and proper size of the reporter should be selected in future study.

### Characterization of the Reporter of ER-PM MCS

**Figure 8:**
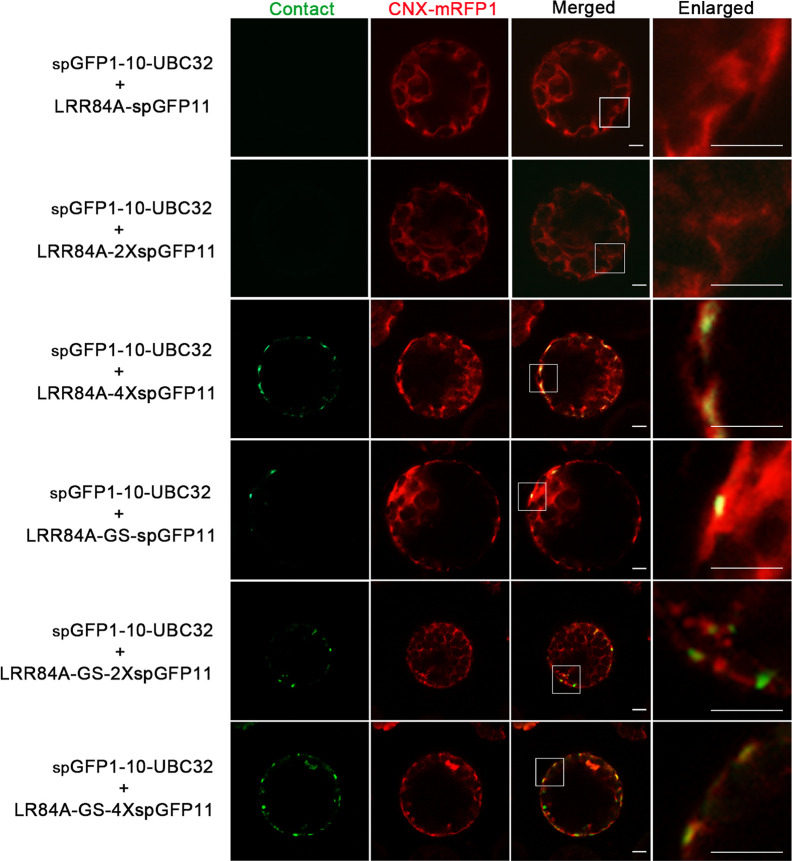
Characterization of the reporter of ER-PM MCS in *Arabidopsis* protoplasts. *Arabidopsis* protoplasts were transiently transformed with a series ER-PM MCSs reporters and CNX-RFP followed by confocal observation. The MCSs were indicated by the green signals; ER was marked by ER marker CNX-RFP. Scale bar: 5 μm.

**Figure 9:**
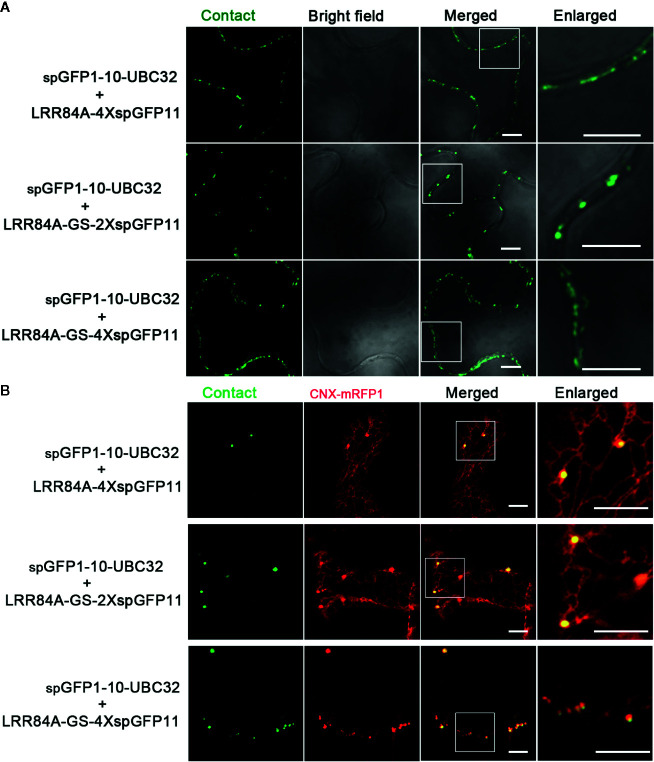
Characterization of the reporter of ER-PM MCS in tobacco (*N. benthamiana*) leaves. **(A)** Tobacco (*N. benthamiana*) leaves were transiently transformed with a series of ER-PM MCSs reporters followed by confocal observation. Scale bar: 5 μm. **(B)** Tobacco (*N. benthamiana*) leaves were transiently co-transformed with a series ER-PM MCSs reporters and the ER marker CNX-mRFP followed by confocal observation. The contact sites were indicated by the green signals; The ER was marked by the ER marker CNX-RFP. Scale bar: 5 μm.

For visualizing the physical association between ER and PM, a series of vectors were introduced into *Arabidopsis* protoplasts following with the same principle described in above. When spGFP1-10-UBC32 was co-expressed with LRR84A-spGFP11 or LRR84A-2XspGFP11 in *Arabidopsis* protoplasts, no fluorescent signal could be observed (**Figure 8**). When the spGFP11 copies increased to four, some bright punctate signals close to the PM were observed and these puncta were also in close proximity to the ER marker CNX-RFP (**Figure 8**). The cells co-expressing spGFP1-10-UBC32 together with either LRR84A-GS-spGFP11 or LRR84A-GS-2XspGFP11 displayed fewer punctate signals when compared with those expressing LRR84A-4XspGFP11. The combinational expression of LRR84A-GS-4XspGFP11 with spGFP1-10-UBC32 created plenty of punctate signals close to PM (**Figure 8**). When expressed in tobacco leaf, these reporters also displayed punctate signals close to PM. Moreover, in comparison with LRR84A-GS-2XspGFP11 and LRR84A-4XspGFP11, the LRR84A-GS-4XspGFP11 produced more punctate signals close to PM when co-expressed with spGFP1-10-UBC32 in tobacco leaf cells (**Figure 9**). When co-expressed with the ER marker CNX-mRFP1, the punctate signals produced from these three ER-PM MCS reporters were found to be closely associated with the ER (**Figure 9B**).

**Figure 10:**
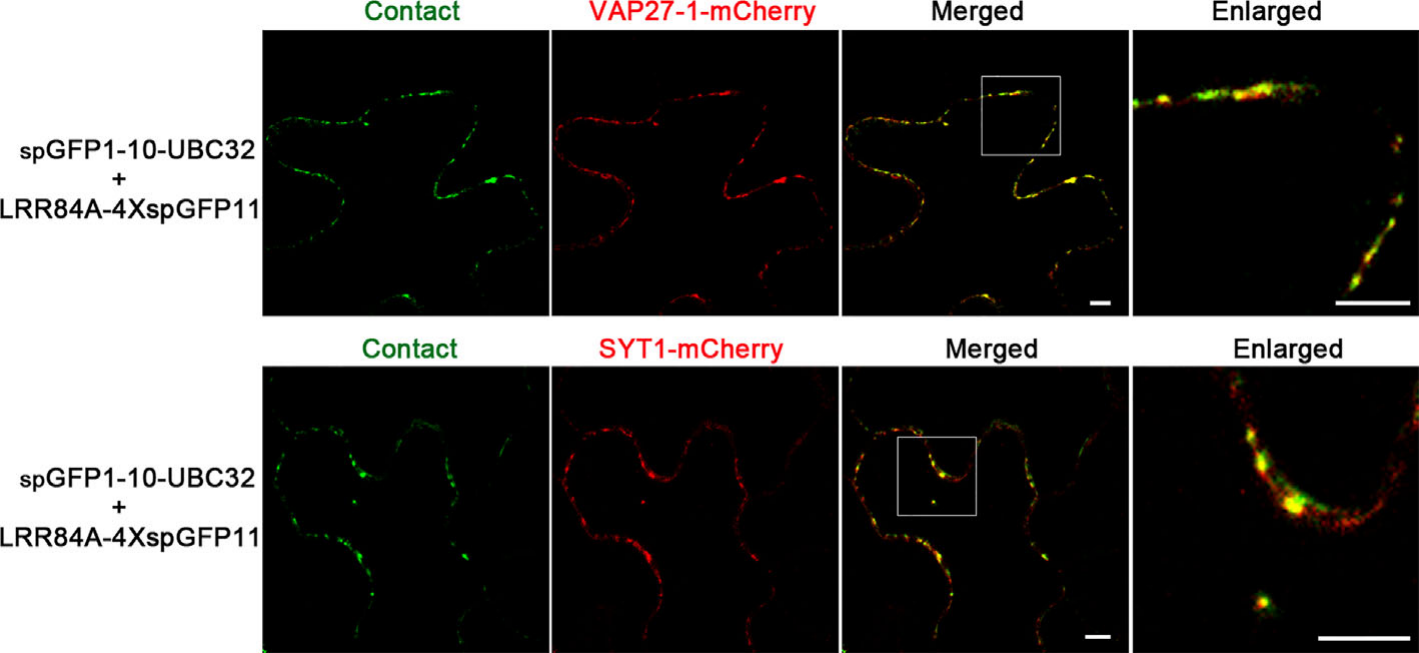
Colocalization of ER-PM MCSs reporter with VAP27-1 and SYT1. Tobacco (*N*. *benthamiana*) leaves were transiently transformed with the ER-PM MCSs reporter and VAP27-1-mCherry or SYT1-mCherry followed by confocal observation. The contact sites were indicated by the green signals. Scale bar: 5 μm.

Compared with other type of ER-based MCSs, the ER-PM MCS is relatively well-characterized in plants. Some protein markers, such as VAP27-1 and SYT1, have been shown to label different populations of the ER-PM MCSs in plants (Wang et al., 2017). To verify whether the ER-PM reporter obtained in this study can label the real ER-PM contact sites, we co-expressed the reporter consisting of LRR84A-4XspGFP11 and spGFP1-10-UBC32 with the known ER-PM MCS markers including VAP27-1-mCherry and SYT1-mCherry. Interestingly, we found that these green puncta of the LRR84A-4XspGFP11 reporter were largely colocalized with both the VAP27-1 and SYT1-labeled ER-PM contact sites, indicating that our reporter might label both populations of ER-PM contact sites in plants (**Figure 10**). Collectively, these results suggest that our obtained reporters can be presumably used to detect ER-PM contact sites in the plants, though caution should be taken in selecting the appropriate spacer length of the reporter.

### Response of ER-Chloroplast MCSs to Cellular Stresses

**Figure 11:**
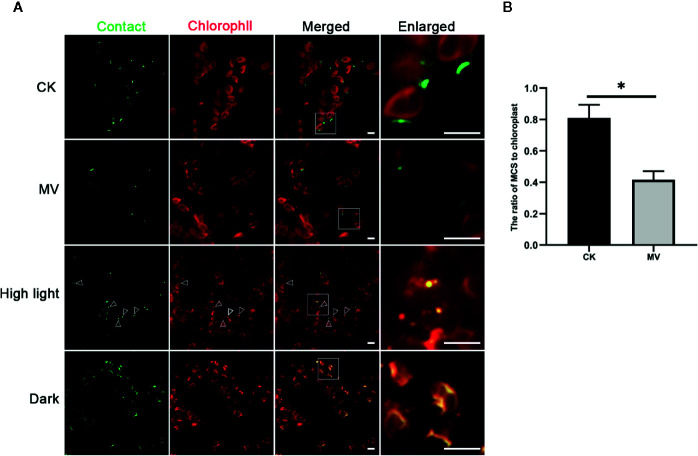
Responses of ER-Chlorolast MCSs to the MV, high light and dark treatments. **(A)** The tobacco (*N. benthamiana*) leaves expressing spGFP1-10-UBC32 and OEP7-4XspGFP11 were infiltrated with 100 μM MV or exposed to high light (1,200 μmol m ^-2^ sec^-1^) and darkness respectively followed by confocal observation. The contact sites were indicated by the green signals; the chloroplast was detected by chlorophyll. Arrowheads indicate the localization of MCSs stained by refold GFP on the punctate dots derived from damaged or fragmented chloroplasts as revealed by chlorophyll autofluorescence. Scale bar: 5 μm. **(B)** Quantification of the density of membrane contact sites in tobacco leaves with or without MV treatment. MCS-to-Chloroplast ratios were calculated by dividing the number GFP-MCSs signal by the number of chloroplast. More than three leaves from two biologically repeated experiments were used for quantification. Asterisk indicates statistically significant difference as determined by student *t*-test.

The ER is a highly dynamic organelle that keeps changing its structure through fusion and fission (Hawes et al., 2015). The chloroplast is also a dynamic organelle, which is the site not only for photosynthesis but also for synthesis of other essential biomolecules like hormones and lipids, thereby actively responding to environmental stresses (Otegui, 2018; Woodson, 2019). Moreover, recent studies in mammal and yeast have shown that MCSs are actively involved in organelle positioning and degradation in response to cellular stresses (Phillips and Voeltz, 2016; Liu and Li, 2019). To investigate the possible dynamics of MCSs in plant, we next choose the ER-chloroplast MCS as an example to explore its possible responses to cellular stresses. We first performed treatment of tobacco leaf expressing spGFP1-10-UBC32 and OEP7-4XspGFP11 with methyl viologen (MV), which is a non-selective herbicide that affects electron transfer kinetics, as a consequence, causes the burst of chloroplastic reactive oxygen species (ROS) and severely damage of chloroplast (Hawkes, 2014; Sétif, 2015). The obtained results demonstrated that the number of MCSs of ER-chloroplast was decreased after MV treatment (**Figure 11**). Previous studies have shown that the mitochondria-ER junctions are involved in ROS signaling communication between ER and mitochondria and the number of MERCs increases with the enhanced level of cellular ROS (Yoboue et al., 2018). In our case, the MV treatment leads to a reduction of ER-chloroplast contact sites. One reason might be due to a different dynamic nature between ER-chloroplast MCS and MERC in response to oxidative stress. Another explanation might be that the mild ROS production in their studies is totally different from the acute burst of ROS due to MV treatment in our study, and such acute burst of ROS exceeds the function of ROS as a signaling molecule but causes strong damage to cellular organelles like chloroplast, thereby disrupting the formation of ER-chloroplast junctions.

We also performed high light treatment and interestingly found that after high light treatment the cells contained few punctate GFP signals that appeared to overlap with the punctate structures derived from damaged chloroplasts as revealed by chlorophyll autofluorescence (**Figure 11**). These punctate structures resemble the rubisco containing bodies (RCBs) or ATI1-containing plastid-associated bodies (ATI-PS Bodies) as observed in previous studies, which are formed in response to carbon starvation or chloroplast damage and are required for transporting a part of stroma or chloroplastic fragments to vacuole for degradation through autophagy pathway (Izumi et al., 2010; Michaeli et al., 2014; Otegui, 2018). These results indicate that the ER-chloroplast MCSs might contribute to the initiation of RCB formation and autophagic degradation of damaged chloroplast. Moreover, the results showed that the GFP signals in leaves under darkness treatment were significantly enhanced (**Figure 11**). These bright GFP signals seemed to wrap the chloroplast and widely distribute inside the cell, suggesting that more frequent communication between ER and chloroplast occurs in darkness or with carbon starvation. Hence, the ER-Chloroplast associations reported by these constructs are dynamic and change with stress (MV and high light) and non-stress (darkness) conditions.

## Discussion

MCSs between membranous organelles in eukaryotic cells have been discovered for decades. Significant progress has been made to characterize the structures, biological functions and maintenance mechanism of MCSs in mammal and yeast. However, the study on plant MCSs is still at the stages of infancy due to the lack of efficient and reliable markers to label MCSs in plant cells. Transmitted electron microscopy has been successfully applied to observe MCSs like ER-PM junction sites in plant, but the usefulness of this conventional approach is limited due to the time-consuming steps and unable to detect MCSs in living cells. The simple colocalization of fluorescent markers among labeled organelles does not always reflect the proximity between these two organelles. Here we design tools to detect plant MCSs in living cells based on fluorescent complementation of spGFP, which is split into two parts spGFP1-10 and spGFP11 and fused to the membrane surfaces respectively. When two organelles or membranes are close enough, spGFP1-10 and spGFP11 come together and resemble as a functional spGFP that emit fluorescence. This split spGFP complementation-based reporter has been successfully used to visualize the MCSs of ER-mitochondria in yeast and mammalian cells (Eisenberg-Bord et al., 2016; Cieri et al., 2018; Kakimoto et al., 2018; Yang et al., 2018).

We demonstrate the usefulness of the split spGFP-based reporters to detect the MCSs of ER-Chloroplast, ER-Mitochondria and ER-PM. Our obtained results showed that the spacer or liner sequences between the split spGFP11 fragment and organelles membrane is critical for correct refolding of spGFP for observation of MCSs. Moreover, the spacer should be modified in different experimental sets. For example, two spGFP11 is enough for generating fluorescent signal to observe potential MCSs of ER-chloroplast, but is too short for observation of membrane junctions between ER and PM. In that case, one or more GS linker can be fused between spGFP11 and membrane anchoring domains to extend the space. These results indicate that the space of MCSs of different organelles might have variable width, a scenario that is indeed supported by evidence obtained by electron microscopes in mammal and yeast (Phillips and Voeltz, 2016). In addition, caution must be taken when repeated GS linker or spGFP11 fragment are used as spacer or booster to enhance the refold GFP signal, because too long of a spacer might exceed the real space between two membranes of MCSs and produce fake fluorescent signals on other membrane junctions besides the real MCSs.

Previous studies have shown that the interaction between the two fragments of split GFP as demonstrated in the commonly used bimolecular fluorescence complementation (BiFC) assay is irreversible *in vitro* (Cabantous et al., 2005; Magliery et al., 2005). Moreover, two recent studies have used the conventional split Venus as BiFC complex to artificially tether the ER membrane to subdomains of PM as well as to tether the multivesicular bodies (MVBs) and tonoplast to the PM (Tao et al., 2019a; Tao et al., 2019b). In their studies, one fragment of Venus (1-155; VenusN) was fused to one integral membrane protein localized on ER or PM and, while another part of Venus (156-239; VenusC) was fused to a MVB or PM localized peripheral membrane protein. When these two parts are overexpressed together in a cell, they will artificially pull two membranes together and create membrane patches that might already undergo membrane fusion (Tao et al., 2019a; Tao et al., 2019b). These artificial tethering effects are largely due to the irreversible assembly of split Venus fragments. In this study, we use split spGFP consisting of spGFP1-10 part and the last β-strand termed spGFP11 fragment, the split site is different from the commonly used split site located in the Venus. Moreover, it has been demonstrated that interaction of spGFP1-10 and spGFP11 can be reversed when used as reporter to detect the MCSs between ER and mitochondria in living mammalian cells (Cieri et al., 2018; Yang et al., 2018). In our study, the MV treatment could also dramatically reduce the formation of fluorescent signals observed in potential MCSs of ER-chloroplast. Moreover, we also demonstrated that the sp-GFP complementation-based reporter designed in this study can successfully label the real ER-PM junctions as revealed by co-expression and colocalization with known markers of ER-PM MCSs in plants. Therefore, those evidence indicate that spGFP1-10 and spGFP11 could be used as a potential reliable system to detect MCSs in living plant cells.

There are a number of studies showing the application of bifluorescence complementation (BiFC) method in detecting MCSs in eukayotic cells (Bockler and Westermann, 2014; Eisenberg-Bord et al., 2016; Harmon et al., 2017; Cieri et al., 2018; Yang et al., 2018). One problem of this method is whether these fluorescent reporters accumulate at pre-existing contact sites or lead to generate new ones. By performing transmission electron microscopic (TEM) analysis of the mammalian cells with or without expression of the split spGFP-based reporter, Yang et al. demonstrated that expression of the ER-mitochondrial MCS reporter did not affect the frequency and the size of the MERCs in the stable cell lines (Yang et al., 2018). More recently, another kind of fluorescently based reporter termed MAPPER (*m*embrane *a*ttached *p*eri*p*heral *ER*) has been developed and used to observe ER-PM junctions in mammalian and plant cells (Chang et al., 2013; Jing et al., 2015; Lee et al., 2019). Total internal reflection fluorescence (TIRF) microscopic analysis showed that expression of the MAPPER reporter did not affect the density of ER-PM junctions (Chang et al., 2013). These results indicate that fluorescent reporter is still suitable for investigating the regulation and functions of MCSs. In our study, we still cannot fully exclude the stable nature of the refolded GFP generated from spGFP1-10 and spGFP11, which might make the reporter less responsive to the stimuli that affect the dynamics of MCSs. In future, immunofluorescent labeling with antibodies against known MCS-labeling proteins or immuno-electron tomography analysis of the plant cells with or without expressing the split spGFP-based reporter will help to address whether it affects the density and morphology of the MCSs in plants. Despite these potential problems, we think that these reporters could be useful tools to detect the spatiotemporal dynamics of MCSs in plant cell at current stage, because an alternative reliable and convenient method to detect MCSs is still lacking in plants. Careful design of the length of spacer, choice of the correct organellar membrane anchoring domains as well as a moderate expression level of the reporter genes are crucial to get a reliable observation of MCSs during the use of these reporters as demonstrated in this study. This fluorescent reporter tool might be especially powerful when used in the large scale forward or reverse genetic screenings to identify potential regulators involved in the regulation of MCSs in plants.

## Data Availability Statement

All datasets generated for this study are included in the article/**Supplementary Material**.

## Author Contributions

CG, TL, and WS designed the project. TL, ZX, HL, and CL performed the experiments. TL, CL, and WS analyzed the results. CG, WS, and TL wrote the manuscript.

## Funding

This work was supported by grants from the National Natural Science Foundation of China (31870171) and Fok Ying-Tong Education Foundation for Young Teachers in the Higher Education Institutions of China (171014) to CG, the National Natural Science Foundation of China (31701246) to WS.

## Conflict of Interest

The authors declare that the research was conducted in the absence of any commercial or financial relationships that could be construed as a potential conflict of interest.

## Supplementary Material

Figure S1 Characterization of the reporter of ER-chloroplast MCS in *Arabidopsis* protoplasts. *Arabidopsis* protoplasts were transiently transformed with different combinations of ER-chloroplast MCSs reporters followed by observation under a fluorescent microscope. The contact sites were indicated by the green signals, red signals were chlorophyll autofluorescence. Scale bar: 10 μm.

Figure S2 Characterization of the reporter of ER-Mitochondria MCS in *Arabidopsis* protoplasts. *Arabidopsis* protoplasts were transiently transformed with different combinations of ER-Mitochondria MCSs reporters followed by observation under a fluorescent microscope. The contact sites were indicated by the green signals, red signals were chlorophyll autofluorescence. Scale bar: 10 μm.
