# Supplementary material for: A Combinatorial Reporter Set to Visualize the Membrane Contact Sites Between Endoplasmic Reticulum and Other Organelles in Plant Cell
Source: Front Plant Sci. 2020 Aug 18;11:1280. doi: 10.3389/fpls.2020.01280 (PMC7461843; doi:10.3389/fpls.2020.01280)
Supplement: Supplementary file 1 [file Table_1.docx]

**[Supplementary Table S1](https://www.sciencedirect.com/science/article/pii/S1674205216300983?via=ihub#mmc1). Primers used in this study.**

| Construct name | Primer name | Primer sequence (from 5’ start to 3’ end) |
| --- | --- | --- |
| pBI221-GFP-UBC32  pBI221-spGFP1-10-UBC32 | UBC32-Forward | GGGACTAGTGCAGCTAGTGTGGTTCCCGCTG |
|  | UBC32-Reverse | gggCTCGAGTCAAGACTGATCATCCATAAACC |
|  | spGFP1-10-Forward | gggGGATCCATGTCCAAAGGAGAAGAACT |
|  | spSPGFP1-10-HAtag-Reverse | GGGACTAGTAGCGTAATCTGGAACATCGTATGGGTATGTTCCTTTTTCATTTGGATC |
| pBI221-OEP7-GFP | OEP7- Forward | gggGGATCCATGGGAAAAACTTCGGGAGCGAAAC |
|  | OEP7-Reverse | GggGGTACCCAAACCCTCTTTGGATGTGGTTG |
| pBI221-OM64-GFP | OM64- Forward | gggGGATCCATGTCGAATACGCTTTCTTTGATTC |
|  | OM64-Reverse | gggGGTACCAGGGAAAGGAAGAAGCTCGAAACG |
| pBI221-LRR84A-GFP | LRR84A-Forward | gggGGATCCATGAAAACAC AATCAGCTTC ACC |
|  | LRR84A-Reverse | gggGGTACCGTTGTTTTTGTATTTATAAACGTAG |
| pBI221-OEP7-spGFP11  pBI221-OM64-spGFP11  pBI221-LRR84A-spGFP11 | GFP11- Forward | gggCTCGAGTGTAATCCCAGCAGCATTTA |
|  | GFP11- Reverse | ggGGTACC GAC TAC AAA GAC GAT GAC GAC AAG ATGCGTGA CCACATGGTCCT |
| pBI221-OEP7-2XspGFP11  pBI221-OM64-2XspGFP11  pBI221-LRR84A-2XspGFP11 | GFP11- Forward | gggCTCGAGTGTAATCCCA GCAGCATTTA |
|  | 2XspGFP11- Reverse | gggCTCGAGTTAAGTGATGCCTGCGGCGTTAAC |
| pBI221-OEP7-4XspGFP11  pBI221-OM64-4XspGFP11  pBI221-LRR84A-4XspGFP11 | GFP11- Forward | gggCTCGAGTGTAATCCCA GCAGCATTTA |
|  | 4XspGFP11- Reverse | gggGGTACCGACTACAAAGACGATGACGACAAGGGATCTGGAGGTTCTAGAGG |
| pBI221-OEP7-GS-spGFP11  pBI221-OM64-GS-spGFP11  pBI221-LRR84A-GS-spGFP11 | GS linker- Forward | gggGGTACCGACTACAAAGACGATGACGACAAGGGATCTGGAGGTTCTAGAGG |
|  | GFP11- Reverse | ggGGTACC GAC TAC AAA GAC GAT GAC GAC AAG ATGCGTGA CCACATGGTCCT |
| pBI221-OEP7-GS-2XspGFP11  pBI221-OM64-GS-2XspGFP11  pBI221-LRR84A-GS-2XspGFP11 | GS linker- Forward | gggGGTACCGACTACAAAGACGATGACGACAAGGGATCTGGAGGTTCTAGAGG |
|  | 2XspGFP11- Reverse | gggCTCGAGTTAAGTGATGCCTGCGGCGTTAAC |
| pBI221-OEP7-GS-4XspGFP11  pBI221-OM64-GS-4XspGFP11  pBI221-LRR84A-GS-4XspGFP11 | GS linker- Forward | gggGGTACCGACTACAAAGACGATGACGACAAGGGATCTGGAGGTTCTAGAGG |
|  | 4XspGFP11- Reverse | gggGGTACCGACTACAAAGACGATGACGACAAGGGATCTGGAGGTTCTAGAGG |
| pBI221-VAP27-1-mCherry | VAP27-1- Forward | TGAGTTTTTCTGATTAACAGGGATCCATGAGTAACA TCGATCTGAT TG |
|  | VAP27-1-Reverse | ACGTCCTCGGAGGAGGCCATCTCGAGTGTCCTCTTCATAA TGTATCCC |
| pBI221-SYT1-mCherry | SYT1- Forward | TGAGTTTTTCTGATTAACAGGGATCCATGAGTAACA TCGATCTGAT TG |
|  | SYT1-1-Reverse | ACGTCCTCGG AGGAGGCCAT CTCGAGTGTC CTCTTCATAA TGTATCCC |
