# Supplementary figures and images for: A Combinatorial Reporter Set to Visualize the Membrane Contact Sites Between Endoplasmic Reticulum and Other Organelles in Plant Cell
Source: Front Plant Sci. 2020 Aug 18;11:1280. doi: 10.3389/fpls.2020.01280 (PMC7461843; doi:10.3389/fpls.2020.01280)

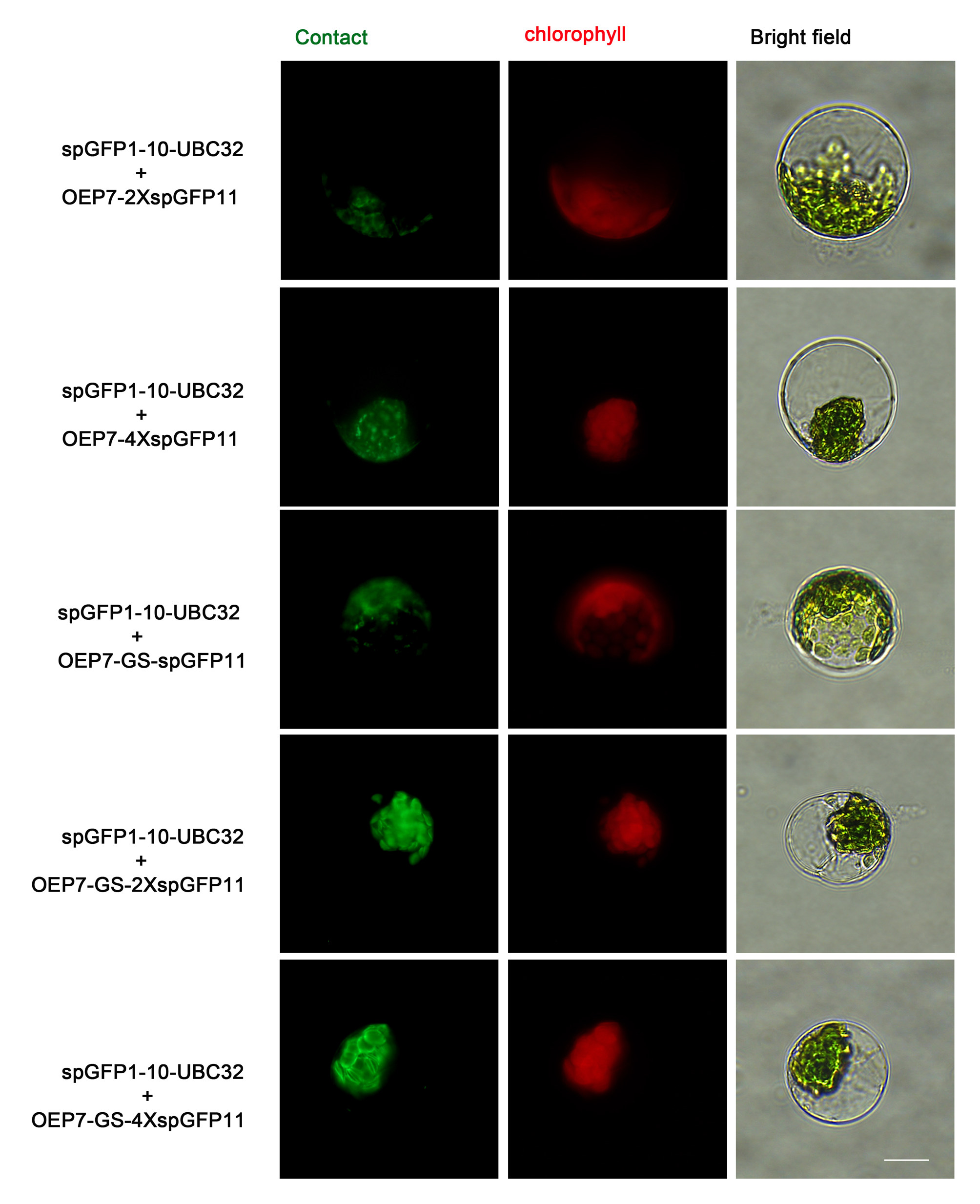

Supplement: Figure S1 — Characterization of the reporter of ER-chloroplast MCS in Arabidopsis protoplasts. Arabidopsis protoplasts were transiently transformed with different combinations of ER-chloroplast MCSs reporters followed by observation under a fluorescent microscope. The contact sites were indicated by the green signals, red signals were chlorophyll autofluorescence. Scale bar: 10 μm. [file Image_1.tif]

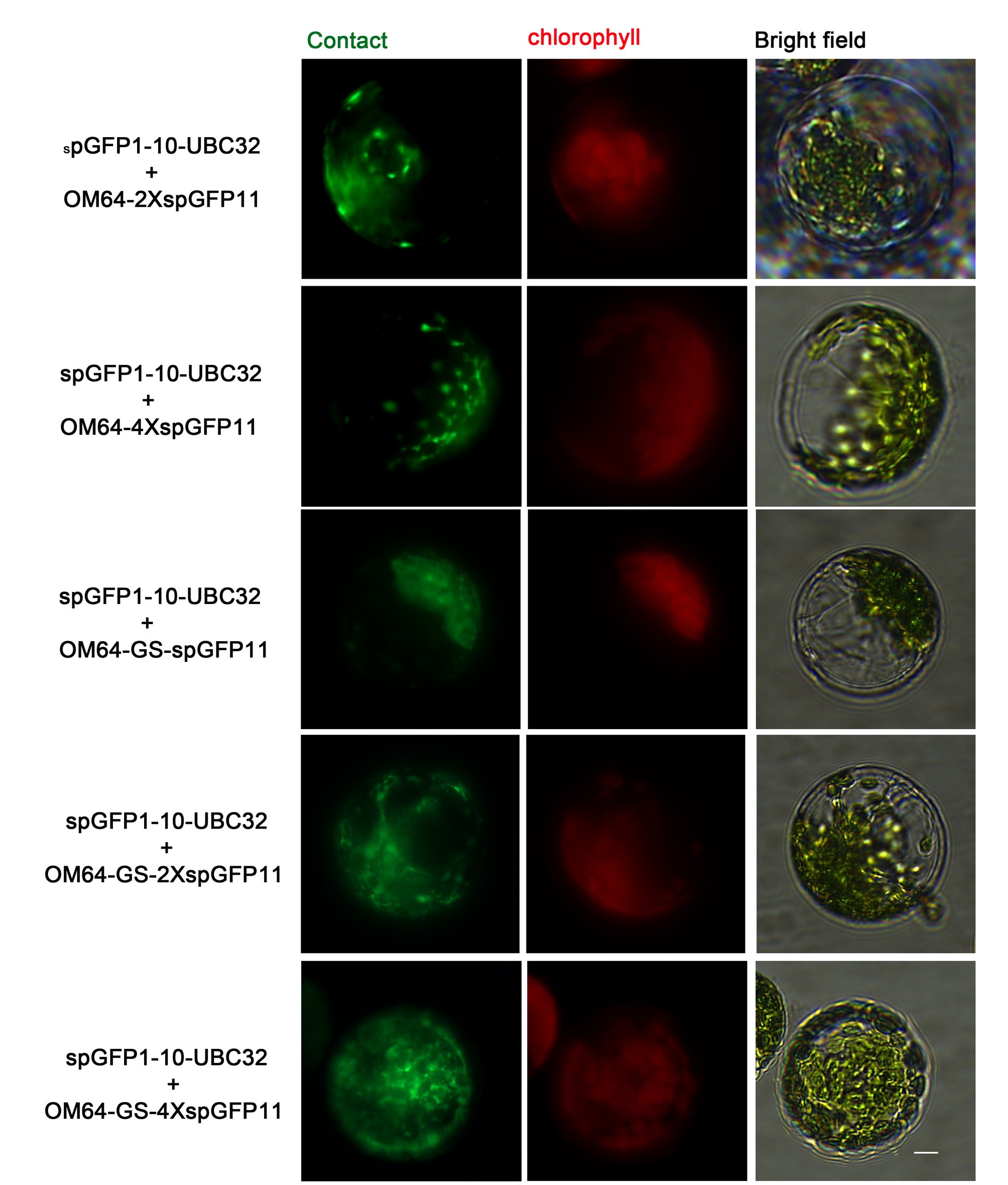

Supplement: Figure S2 — Characterization of the reporter of ER-Mitochondria MCS in Arabidopsis protoplasts. Arabidopsis protoplasts were transiently transformed with different combinations of ER-Mitochondria MCSs reporters followed by observation under a fluorescent microscope. The contact sites were indicated by the green signals, red signals were chlorophyll autofluorescence. Scale bar: 10 μm. [file Image_2.tif]
